# Uncovering the influence of nitridation on the dislocation density at atomistic scale in III-Nitrides MOCVD/MOVPE epitaxy process

**DOI:** 10.1038/s41598-025-89681-y

**Published:** 2025-04-12

**Authors:** P. K. Saxena, P. Srivastava, Anshika Srivastava, Anshu Saxena

**Affiliations:** Tech Next Lab Pvt Ltd, Lucknow, UP India

**Keywords:** Hetero-epitaxy, Atomistic, TNL-TCAD, Simulation, Defects, TNL-EpiGrow, III-Nitrides, GaN, AlN, Materials for devices, Theory and computation, Atomistic models, Computational methods

## Abstract

The impact of NH_3_ pre-flow duration on the strain development of AlN and its alloy buffer layers, as well as GaN layers, was reproduced through atomistic simulation. The reported method provides access to information that is not obvious from the raw data alone. The growth morphology of AlN utilizing pre-nitridated silicon substrates was analyzed with respect to the mechanisms of defect formation in each deposited monolayer. This report presents two distinct case studies concerning the epitaxy of the III-nitrides. In the first case study, the crystalline quality of AlN deposited on silicon substrates with NH_3_ pre-flow durations of 0 s and 30 s was compared. The growth rates of the samples were aligned with those from previous simulation studies published by our group. It was noted that the defect density extracted from the sample with a 30-second NH_3_ pre-flow was lower than that of the sample with a 0-second pre-flow. The results obtained from this preliminary case study led to a repetition of the multistep hetero-epitaxy experiment, previously reported by Kadir et al., with modifications to the NH_3_ pre-flow duration on the silicon substrate in the subsequent case study. The epitaxial growth of GaN on AlN and three-step graded Al_x_Ga_1−x_N (where x = 0.8, 0.5, and 0.2) strain relief layers was simulated at the atomistic scale using the MOCVD process over silicon (111) substrates, with variations in NH_3_ pre-flow times. The strain induced by lattice mismatch between the silicon substrate (both without and with NH_3_ pre-flow) and the various buffer layers was examined in terms of dislocation density extracted layer-by-layer. The effect of NH_3_ pre-flow time on the generation of threading dislocation density (TDD) in each monolayer of the AlN and AlGaN buffer layers was analyzed. It was determined that the duration of NH_3_ pre-flow significantly influences the morphology and quality of each deposited monolayer justifies the experimental observations. A higher likelihood of amorphous SiN_x_ formation was observed with no and shorter NH_3_ pre-flow times. The lowest TDDs across all strain relief layers were measured at approximately ~ 10^10^ cm^− 2^. The dislocations generated in the initial buffer layer (AlN) were identified as contributing to the TDD in the subsequent layers. Furthermore, it was noted that the sample lacking an intentional nitridation step displayed a higher TDD and vacancy density compared to those with optimal nitridation.

## Introduction

The silicon substrate has emerged as the most suitable choice for the growth of GaN and its alloys, primarily due to its affordability, superior material quality, high thermal conductivity, and availability in large diameters, making it ideal for various industrial and technological applications compared to alternatives such as sapphire or SiC substrates. The well-established integrated circuit technology associated with silicon substrates renders them the most economically viable option for the advancement of III-Nitride based electronic and optoelectronic devices in industrial settings^1^. However, the significant lattice mismatch results in a high dislocation density and the differing material properties between GaN and silicon present several challenges during the epitaxial growth of GaN on silicon. The thermal expansion coefficient (TEC) mismatch at room temperature is approximately 53%. This mismatch varies at elevated growth temperatures, leading to the formation of cracks in the GaN film due to substantial tensile stress during the growth process^2^. Additionally, gallium acts as a potent etchant for silicon, a phenomenon known as melt-back etching^3^. To mitigate strain, buffer layers of AlN and Al_x_Ga_1−x_N are employed between the silicon and GaN layers. When GaN is grown on Al_x_Ga_1−x_N, compressive strain is induced due to the smaller in-plane lattice constant of AlN. This strain in GaN is balanced by the strain that develops in silicon as a result of the temperature differential between the two materials during the cooling process from growth temperature to room temperature. Over the past three decades, extensive research has been conducted by scientists exploring various buffer layers, alloy compositions and the effects of temperature and pressure^4–10^. However, understanding the nucleation process of the AlN layer on silicon substrates has proven challenging for experts on a case-by-case basis. Observations indicate that the quality of the AlN layers has a significant impact on the stress present in the final GaN layer, despite their nominally identical characteristics^9^. Hetero-epitaxial growth presents a promising avenue, albeit with several challenges such as lattice mismatch, strain, and various types of defect generation.

Two primary strategies are employed to reduce strain in the epitaxy of III-Nitrides on Si (111) substrates: pre-nitridation or pre-aluminization prior to the deposition of the buffer layer^9^. It has been noted that the concurrent flow of TMAl and NH_3_ increases the likelihood of forming amorphous SiN_x_ patches, which can be mitigated by employing a TMAl predose. Nevertheless, in many instances, the unintended rapid formation of SiN_x_ could not be entirely prevented^2,11^. The investigation of a separate nitridation step before introducing TMAl into the reactor has also been conducted^12–17^, resulting in a substantial decrease in the occurrence of SiN_x_ patches. The likelihood of SiN_x_ patch formation (amorphous islands) is influenced by the surface temperature during the simultaneous flow of TMAl and NH_3_. In contrast, when employing an optimally timed pre-flown NH_3_ substrate, the entire surface of the substrate is enveloped by a Si_3_N_4_ layer, resulting in a minimal chance of additional nitrogen diffusion into the silicon substrate and amorphous SiN_x_ patches formation. However, the optimal NH_3_ pre-flow duration and pre-nitridation temperature remain subjects of debate for achieving high-quality GaN films on Si. Prior experimental investigations have effectively shown the impact of NH_3_ pre-flow duration on the Si substrate^18,19^. Nevertheless, there are numerous intricate and challenging problems related to the epitaxy of III-nitride compounds^20^, necessitating extensive experimentation.

The aim of this study is to illustrate that the MOCVD/MOVPE processes for III-nitrides can be effectively modeled through atomistic simulations, allowing for precise predictions and consistent replication of the growth phenomena observed in actual reactor settings^21–27^. This work presents a cost-effective approach to minimizing raw material usage, shortening process development timelines, and reducing manpower requirements in comparison to the costly III-V nitrides epitaxy process, as detailed herein^21–27^. The TNL-EpiGrow™ simulator has the capacity to simulate billions of atoms, contingent upon the specifications of the system hardware utilized for the simulation. A trade-off exists between the number of atoms simulated and the capabilities of the system hardware. Due to its atomistic nature, the simulator requires substantial memory (RAM) and hard drive space. Currently, it is capable of identifying point defects such as vacancies and interstitials, as well as line defects like edge dislocations. The authors are actively engaged in enhancing the simulator’s functionality to also extract screw dislocations and stacking faults.

This study examines the impact of NH_3_ pre-flow and its duration through atomistic growth simulations, complemented by experimental validation to uncover more detailed insights. All simulations were conducted using the TNL-EpiGrow™ simulator. The initial section of the manuscript presents a comparative analysis of growth morphology between two samples, focusing on AlN epitaxy simulations performed on both non-pre-nitridated and pre-nitridated Si (111) substrates. In the subsequent section, a multi-steps hetero-epitaxy simulation was carried out to replicate the findings of an experiment^19^. This six-step epitaxy process included low temperature (LT) AlN, high temperature (HT) AlN, Al_x_Ga_1−x_N (x = 0.2, 0.5, 0.8) layers, culminating in GaN to produce a high-quality film on Si (111) substrates. The strain depends on the mismatch of the lattice constants as well as on the substrate orientation. The study investigated the effects of varying NH_3_ pre-flow times—specifically 0 and 30 s—along with intervals of 0, 5, and 30 s on the Si substrate and their influence on different strain relief layers. Three samples were grown with systematic variations in NH_3_ pre-flow time. The growth rates of each strain relief layer were compared to the thickness measurements from the experiment^19^. Additionally, the effects of pre-nitridation time on each deposited monolayer were analyzed in terms of threading dislocation density (TDD) and vacancy density.

## Atomistic method

The in-house developed TNL-EpiGrow™ simulator was employed to model the epitaxial growth of all samples in the two case studies. This simulator offers a cost-efficient means to optimize the input parameters related to the epitaxial growth processes across various material systems. Depending on the specific needs of the user, the geometries of vertical and horizontal flow MOCVD reactors from Aixtron, Veeco, and Taiyo Nippon can be integrated into the TNL-EpiGrow™ simulator^20^. Users are required to provide real-time reactor process input parameters, including reactor geometry, substrate specifications such as size, system type, orientation, and temperature, the number of precursors, various energy barrier values, carrier gas details along with their flow rates, chamber pressure, and specifics of gas- and surface-phase chemical kinetic reactions. No coding or scripting is necessary to execute the growth process simulation. The gas- and surface-phase chemical kinetics are managed through built-in algorithms that utilize entropy and enthalpy. The seven coefficient values for calculating entropy and enthalpy are sourced from the National Institute of Standards and Technology (NIST) database^21^. The TNL-chemical kinetics utility package simulates the kinetics of gas and surface phase chemical reactions based on reactor geometry and input conditions. Additionally, it allows for the estimation of the flux related to Arrhenius parameters of each reaction. The chemical reactions pertinent to the AlN material system have been detailed in previously published works by our research group^22,23^. The rates of adsorption, hopping, and desorption events occurring at the substrate surface are determined using a kinetic Monte Carlo algorithm that incorporates randomness, mirroring the natural deposition processes observed in actual metalorganic chemical vapor deposition (MOCVD) reactors^22–25^. The methodology employed throughout the deposition process leverages the dependence of coupled reactor geometry parameters on chemical kinetics, as well as the mechanisms of adsorption, hopping, and desorption, to accurately replicate real-time MOCVD reactor epitaxy experiments via atomistic simulation. This is elaborated upon in the following sections. In this study, the Aixtron Crius^®^ close-coupled-shower head (CCS) reactor geometry is considered for the epitaxy simulation.

### Reactor geometry

The impact of the geometry of the showerhead MOCVD reactor is considered by utilizing a showerhead flange equipped with numerous small holes of radius r, which directs the reactant gases vertically onto the substrate. The vertical distance between the showerhead and the substrate is defined as the ceiling height, H_C_, while the radius of the reaction chamber, R, is employed to calculate the residence time (t_res_) is^28^,

1 $$\:{t}_{res}=-\frac{{H}_{c}}{{v}_{in}}ln\frac{\delta\:}{{H}_{c}}+\frac{{\delta\:}^{2}}{4D}$$

In stagnation flow, the boundary layer thickness, denoted as δ, is influenced by the velocity v_in_, which is a function of the radius of the small holes (r). The radius of the chamber is taken into account in term of ratio of the diffusion length and the chamber radius $\:\left({L}_{d}/R\right)$, which defines the radial mass transport, while the ratio of the diffusion length and the ceiling height $\:\left({L}_{d}/{H}_{C}\right)$ is considered for axial (vertical) mass transport of the reactor.

The radius of the showerhead is taken into account in term of vertical velocity. Two components of gases velocity are considered inside chamber i.e. radial velocity, $v_{r} = v_{{in}} \frac{r}{{2H_{C} }}$ and vertical velocity, $\:{v}_{z}={v}_{in}\left(-\frac{z}{{H}_{C}}\right)$. The both vertical and radial velocities are considered linear in height and in radius respectively. The total fluid velocity is $\:\left|v\right|=\frac{{v}_{in}}{2{H}_{C}}\sqrt{{r}^{2}+{4z}^{2}}$, Here, v is the velocity vector; v_z_ the vertical velocity; v_in_ the inlet velocity; H_C_ the ceiling height. The term $\:\frac{{{\updelta\:}}^{2}}{4\text{D}}$ in Eq. (1) indicates the diffusion time derived from the diffusion length while D represents the mass diffusivity. The residence time, $\:{\text{t}}_{\text{r}\text{e}\text{s}}$, is proportional to $\:\frac{{\text{H}}_{\text{c}}}{\text{v}}$, with a slowly varying logarithmic factor as a multiplier. Notably, the boundary layer thickness δ remains constant across different radial positions, meaning that the effective residence time $\:{\text{t}}_{\text{r}\text{e}\text{s}}$ is solely a function of height and does not vary with radial position. The estimation of the boundary layer thickness in stagnation flow is referenced in^28^.

2 $$\:\frac{\delta\:}{{H}_{c}}=3\sqrt{\frac{\pi\:{R}^{2}{\gamma\:}_{STP}}{{V}_{m}{F}_{in}{H}_{c}}}$$

In this context, γ_STP_ represents kinematic viscosity, F_in_ denotes the molar flow, and V_m_ indicates the molar volume at standard temperature and pressure (STP). According to Eq. (2), it is clear that:

- For $\:\frac{{\updelta\:}}{{\text{H}}_{\text{c}}}$≥ 1, convection has a minimal impact on the vertical transport of mass and momentum.
- For $\:\frac{{\updelta\:}}{{\text{H}}_{\text{c}}}$ ≤ 1, the boundary layers are relatively thin in comparison to the height of the chamber, resulting in stagnation flow.
- If both the flow rate and ceiling height are increased, convection will become increasingly significant in the vertical transport process.

### Chemical kinetics

Table 1, illustrates the probabilities associated with different reversible and irreversible chemical reactions, as well as the reaction pathways occurring in both the gas phase and surface phase during the MOCVD process. The forward rate constants for the ith reaction are determined using a modified Arrhenius equation^20^.

3 $$\:{k}_{fi}=A{T}^{n}\text{e}\text{x}\text{p}\left(-\frac{{E}_{a}}{{R}_{G}T}\right)$$

In this context, E_a_ (calorie) represents the activation energy, while the coefficient A, referred to as the pre-frequency factor, is influenced by the entropy of formation. The prefactor A, which has units that vary based on the reaction order, encompasses details about the random motion of molecules as they undergo vibration, rotation, and translation within the reactor environment, contributing to the calculation of the molecules’ thermal energy. The operating temperature is denoted by T, n signifies the temperature exponent, and R_G_ is the universal gas constant, which is applicable solely in relation to the activation energy E_a_ and maintains consistent units^21^.

The reverse rate constants $\:{\text{k}}_{\text{r}\text{i}}$ are dependent on the forward rate constants through the equilibrium constants as^20–24^;

4 $$\:{\text{k}}_{\text{r}\text{i}}=\frac{{\text{k}}_{\text{f}\text{i}}}{{\text{K}}_{\text{c}\text{i}}}$$

The thermodynamic properties in pressure units^21^;

5 $$\:{\text{K}}_{\text{c}\text{i}}={\text{K}}_{\text{p}\text{i}}{\left(\frac{{\text{P}}_{\text{a}\text{t}\text{m}}}{{\text{R}}_{\text{G}}\text{T}}\right)}^{{\sum\:}_{\text{k}=1}^{\text{K}}{{\upnu\:}}_{\text{k}\text{i}}}$$

**Table 1:** Gas phase and surface reactions.

Reactants		Products	
Gas phase equations			
Al(CH_3_)_3_	$\:\rightleftharpoons\:$	AlCH_3_ + 2CH_3_	
Al(CH_3_)_3_+NH_3_	$\:\rightleftharpoons\:$	Al(CH_3_)_3_:NH_3_	
Al(CH_3_)_3_:NH_3_	$\:\rightleftharpoons\:$	Al(CH_3_)_3_+NH_3_	
Al(CH_3_)_3_:NH_3_	$\:\rightleftharpoons\:$	(CH_3_)_2_Al: NH_2_ + CH_4_	
Al(CH_3_)_3_:NH_3_ + NH_3_	$\:\rightleftharpoons\:$	(CH_3_)_2_Al: NH_2_ + CH_4_ + NH_3_	
2(CH_3_)_2_Al: NH_2_	$\:\rightleftharpoons\:$	((CH_3_)_2_Al: NH_2_)_2_	
Gas to surface phase equations			
Al(CH_3_)_3_+ vacancy site	$\:\rightleftharpoons\:$	Al(S) + 3CH_3_	
Al(CH_3_)_3_:NH_3_ + vacancy site	$\:\rightleftharpoons\:$	Al(S) + 3CH_3_ + NH_3_	
AlCH_3_ + vacancy site	$\:\rightleftharpoons\:$	Al(S) + CH_3_	
(CH_3_)_2_Al: NH_2_ + vacancy site	$\:\rightleftharpoons\:$	AlN(S) + 2CH_4_	
((CH_3_)_2_Al: NH_2_)_2_+ vacancy site	$\:\rightleftharpoons\:$	2AlN(S) + 4CH_4_	

Here, (S) signifies the details of adsorbed atoms/molecules at the surface.

$\:{\text{K}}_{\text{c}\text{i}}$ is the equilibrium constant in concentration units and easily determined from the Eq. (5), where, $\:{\text{P}}_{\text{a}\text{t}\text{m}}$ denotes a pressure of 1 atm and $\:{{\upnu\:}}_{\text{k}\text{i}}$ is the stoichiometric coefficients and taken as integers. The equilibrium constants $\:{\text{K}}_{\text{p}\text{i}}$ are obtained through thermodynamic properties as;

6 $$\:{\text{K}}_{\text{p}\text{i}}=\text{e}\text{x}\text{p}\left(\frac{{\Delta\:}{\text{S}}_{\text{i}}^{\text{o}}}{{\text{R}}_{\text{G}}}-\frac{{\Delta\:}{\text{H}}_{\text{i}}^{\text{o}}}{{\text{R}}_{\text{G}}\text{T}}\right)$$

Here, $\:{\Delta\:}$ refers to the change that occurs in passing completely from reactants to products in the i^th^ reaction. The computation of enthalpy ($\:\varDelta\:H$) and entropy ($\:\varDelta\:S$) are based seven coefficients using NIST chemical equilibrium data in the form of polynomial fits^21^.

The difference of forward rates ($\:{\text{k}}_{\text{f}\text{i}}$) and the reverse rates ($\:{\text{k}}_{\text{r}\text{i}}$) of reversible reaction generates the progress variable Q_i_ for the i^th^ reaction;

7 $$\:{\text{Q}}_{\text{i}}={\text{k}}_{\text{f}\text{i}}\prod\:_{\text{k}=1}^{\text{K}}{\left[{\text{X}}_{\text{k}}\right]}^{{{\upnu\:}}_{\text{k}\text{i}}^{{\prime\:}}}-{\text{k}}_{\text{r}\text{i}}\prod\:_{\text{k}=1}^{\text{K}}{\left[{\text{X}}_{\text{k}}\right]}^{{{\upnu\:}}_{\text{k}\text{i}}^{{\prime\:}{\prime\:}}}$$

Here $\:\left[{\text{X}}_{\text{k}}\right]$ is the molar concentration of $\:{\text{k}}^{\text{t}\text{h}}$ species, $\:{{\upnu\:}}_{\text{k}\text{i}}$ is the stoichiometric coefficients and taken as integers.

### Deposition process

The TNL-EpiGrow simulator incorporates the kinetic Monte Carlo (KMC) algorithm, which calculates the overall rate resulting from adsorption, diffusion, and desorption processes^20^.

8 $$\:{\text{R}}_{\text{d}\text{e}\text{p}}=\text{A}+\text{H}+\text{D}$$

In this context, $\:{\text{R}}_{\text{d}\text{e}\text{p}},$ is the total rate, A, H, and D represent the rates of absorption, diffusion, and desorption, respectively, as outlined in previously published studies from our research group^22–27^. The surface activation energy is crucial for determining that bonds are formed solely between adjacent atoms. This energy varies for atoms across different material systems. Consequently, the overall surface activation energy is influenced by the surface diffusion energy barrier, the nearest binding energy, and the number of nearest neighbors (nn) present on the surface^20^.

9 $$\:E={E}_{s}\pm\:n{E}_{n}$$

Here, E_s_ and E_n_ denote the energy barriers for surface diffusion and the nearest binding energy, respectively. The variable n indicates the count of nearest neighbors (nn) present on the surface. The influence of step-edge barriers is accounted for by considering descending steps, commonly referred to as the Schwoebel barrier, and ascending steps, known as the incorporation barrier, throughout the surface diffusion process. Consequently, the activation energy required for an atom is:

10 $$\:E=\left\{\begin{array}{c}{\text{E}}_{\text{s}}+n{\text{E}}_{\text{n}}+{\text{E}}_{\text{s}\text{h}\text{w}}\\\:{\text{E}}_{\text{s}}+n{\text{E}}_{\text{n}}+{\text{E}}_{\text{i}}\end{array}\right.$$

where, E_shw_ and E_i_ denote the Schwoebel and incorporation barriers energies respectively. These two barriers are destination dependent, so the activation energy for the same atom is also dependent on diffusion destination.

## Results and discussion

This section focuses on the findings derived from two distinct case studies. The substrate orientation selected for both case studies is Si(111). Indeed, the in-plane and vertical lattice parameters of the substrate (a and c), as well as its orientation, play a crucial role in influencing the growth morphology and various other outcomes. Within the TNL-EpiGrow simulator, substrate orientations of < 100>, < 110>, and < 111 > can be selected; however, (111) oriented silicon wafers are predominantly utilized for GaN epitaxy, as they effectively mitigate the tensile stress in the GaN layer, facilitating the growth of crack-free GaN films.

The first case study illustrated a simulation of AlN epitaxial growth, utilizing the MOCVD process on square-shaped Si (111) substrates measuring 200 × 200×(unit cell)^2^, approximately ≈ 0.0059 µm^2^ (with a unit cell of Si equating to 3.84 Å). TMAl and NH_3_ served as the precursors. A comparison was made between the growth morphologies of AlN deposited on substrates without and with pre-nitridation. The analysis included the extraction of defect generation caused by strain in each monolayer of both samples to assess differences in growth morphology. The input data is tabulated in Table 2 and extracted output data of first case study is shown in Table 3. The growth rates of each sample were corroborated with experimental data^23^. In the second case study, the authors successfully replicated a high-quality GaN hetero-epitaxy process through simulation, as referenced in^19^. This involved the use of two-step AlN and three-step graded Al_x_Ga_1−x_N strain relief layers on the Si (111) substrate, with variations in NH_3_ pre-flow times as outlined in Table 4. Three samples, designated A, B, and C, were grown on the Si substrate with NH_3_ pre-flow times of 0s, 5s, and 30s, respectively. The film morphology and defect generation mechanisms were analyzed across the three samples. The thickness of each strain relief layer, including AlN, Al_0.8_Ga_0.2_N, Al_0.5_Ga_0.5_N, Al_0.2_Ga_0.8_N, and GaN, was validated against experimental results under identical input conditions as described in^19^.

The total pressure within the chamber was maintained at a constant level throughout each scenario of the deposition simulation. The viscosity and mass diffusivity of TMGa and NH_3_ were utilized to calculate the residence time of the compounds for each material based on gas-phase kinetics. It was noted that various energy barriers, including nearest neighbor, surface, Schwoebel, incorporation, and desorption energy, significantly influenced atom bonding and the growth mode. These factors govern the surface kinetics. The values employed as fitting parameters were optimized to accurately reflect the experimental growth morphology and rates.

**Fig. 1:**
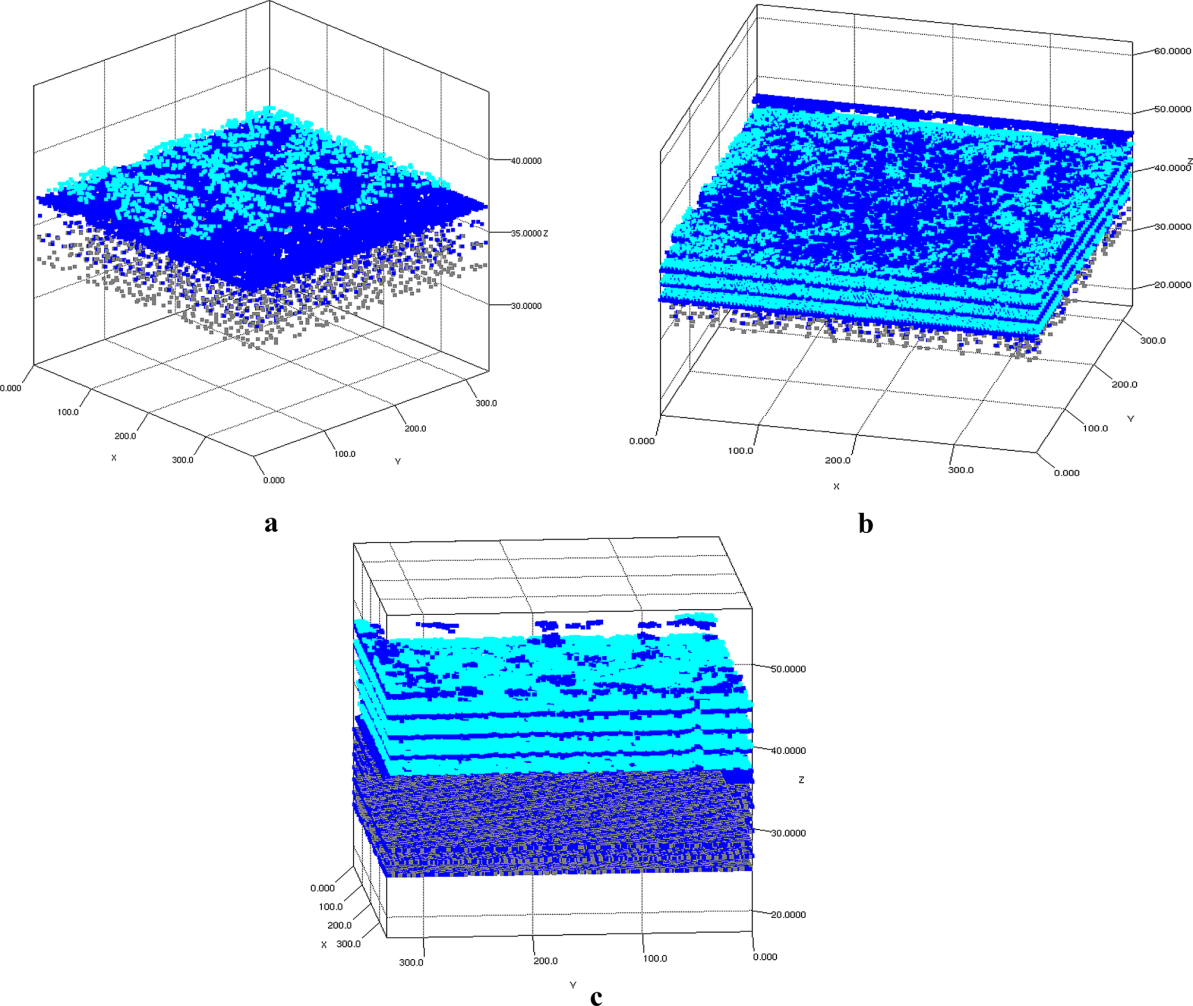
The growth morphology profiles justify the Stranski−Krastanov (SK) growth mode for (**a**) 1ML-AlN on Si(111) with an NH3 pre-flow duration of 0 seconds, (**b**) 4ML-AlN on Si(111) with an NH3 pre-flow duration of 5 seconds, and (**c**) 4ML-AlN on Si(111) with an NH3 pre-flow duration of 30 seconds. (Gray squares represent Si atoms, blue squares denote N atoms, and cyan squares indicate Al atoms).

Prior to discussing the results from each case study, an investigation into the types of growth modes was conducted. The growth profiles for deposited single monolayer (1ML), 4ML on silicon surfaces, and 4ML of AlN on pre-nitridated silicon surfaces were analyzed, as illustrated in Fig. 1a,b,c, respectively. In these figures, silicon, nitrogen, and aluminum atoms are represented by gray, blue, and cyan squares, respectively. Figure 1a,b clearly indicate that the likelihood of nitrogen atoms forming bonds with silicon atoms is significantly higher than that with aluminum atoms when both TMAl and NH_3_ precursors are simultaneously introduced onto the silicon substrate. It was noted that nitrogen atoms diffused into the silicon substrate, resulting in the formation of amorphous SiN_x_ patches. The non-uniform embedding of nitrogen atoms (blue squares) within silicon substrate (gray squares) is evident in Fig. 1a,b, supporting the experimental findings regarding diffusion and the probability of SiN_x_ patch formation. Once a nitrogen atom was deposited on the silicon surface, aluminum atoms began to bond with the underlying nitrogen atoms. The distribution of deposited aluminum atoms (cyan squares) did not exhibit a consistent growth pattern; instead, aluminum atoms were deposited randomly across the surface, leading to the formation of islands of aluminum atoms. Similar growth patterns were noted in the nucleation of AlN on pre-nitridated Si surfaces, with the exception of the uniform distribution of nitrogen atoms (represented as blue squares) within silicon atoms (depicted as gray squares), which supports the formation of the Si_3_N_4_ phase. This observation also corroborates the experimentally determined growth mode and morphology. Initially, the growth proceeded in a three-dimensional (3D) manner, characterized by densely packed, nano-sized island formation up to a specific critical thickness influenced by lattice mismatch, a process known as Volmer-Weber growth mechanisms. Upon reaching this critical thickness and minimizing strain, a transition in the growth mode occurs, leading to a two-dimensional (2D) growth phase known as Stranski − Krastanov (SK) growth mode, where layer-plus-island growth mode deposition takes place. This transition is driven by minimized bonding energies with neighboring atoms. Strain relaxation occurs at the surfaces of 3D islands, which have high surface-to-volume ratios.

**Table 2:** Input conditions used in simulation of Ist case study part I.

Parameters	Si/AlN	Pre-nitridated Si/AlN
Chamber pressure (mbar)	40	40
Shower hole diameter (cm)	0.6	0.6
Chamber radius (cm)	15	15
Chamber volume (L)	1.4	1.4
Ceiling height (cm)	1	1
Substrate temperature (°C)	1050	1050
Precursors	TMAl& NH3	TMAl& NH3
V/III ratios	10	10
Carrier gas	H_2_	H_2_
Nitridation temperature (°C)	–	1050
Nitridation time (s)	–	30
Surface energy (eV)	1.5-2.0	1.5-2.0
Desorption barrier (eV)	3.0	3.0
Schwoebel barrier (eV)	0.05-0.15	0.05-0.15
Incorporation barrier (eV)	0.05-0.15	0.05-0.15
Nearest neighbour (eV)	0.05-0.15	0.05-0.15
No. of interactive elements	1	1

**Table 3:** Extracted outputs of Ist case study in part I.

Parameters	AlN/ Si	AlN/Pre-nitridated Si
Si_3_N_4_ thickness (nm)	–	3.7817
Total deposited atoms (AlN)	5046178	5233815
Vacancies (Å^−3^)	36069	13393

**Table 4:** Pre-nitridation time of IInd case study of part II.

Sample	Nitridation time	Nitridation temp (°C)	Chamber pressure
A	0	995	10^4^ (Pa)
B	5	995	
C	30	995	

### Section I: effect of NH_3_pre-flow on TDD generation

**Fig. 2:**
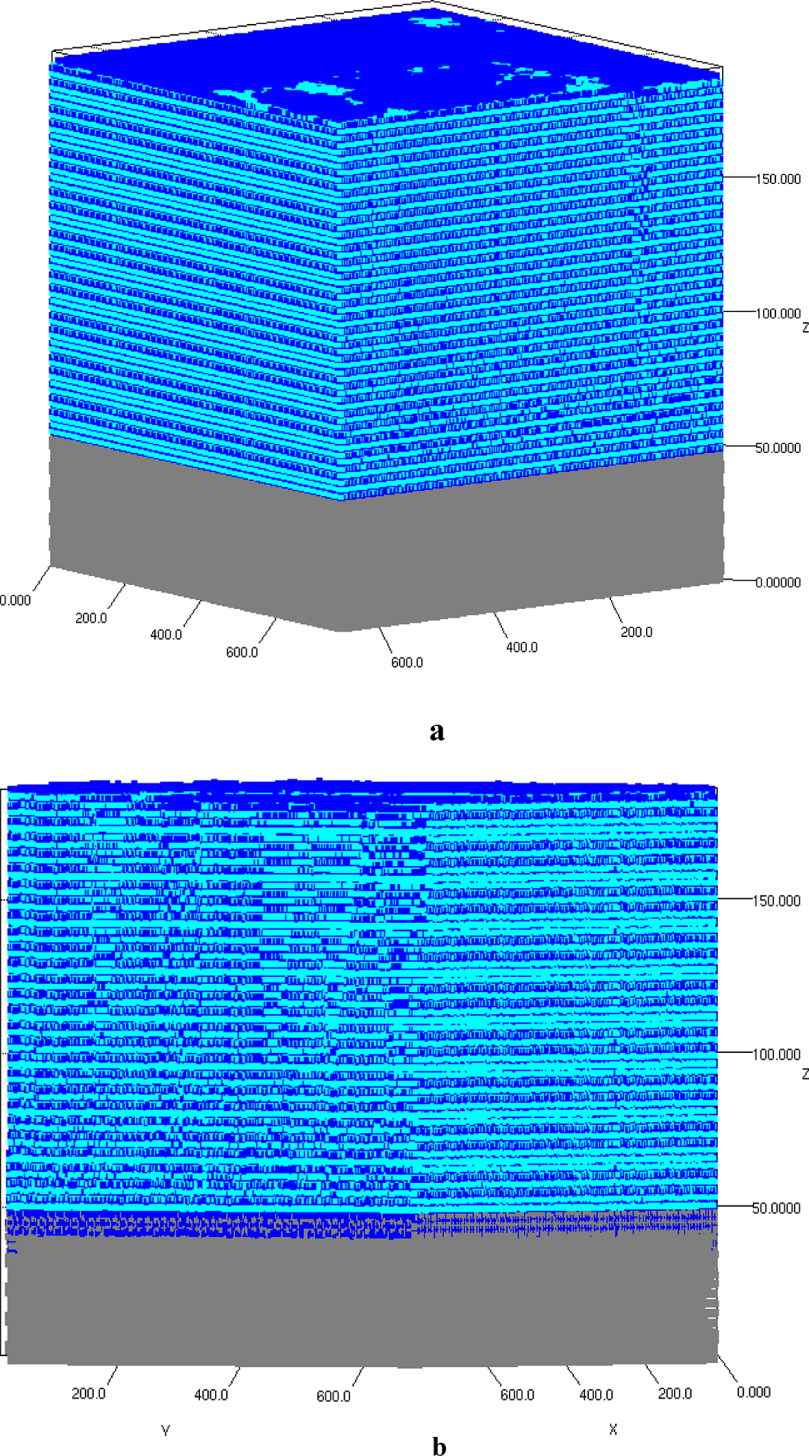
A schematic 3D atomic representation of the structure developed through simulation: (**a**) AlN on Si(111) with an NH_3_ pre-flow duration of 0 seconds; (**b**) AlN on Si(111) with an NH_3_ pre-flow duration of 30 seconds. The squares in shades of gray, blue, and cyan symbolizes silicon, nitrogen, and aluminum atoms, respectively.

The input parameters utilized in the present case study are outlined in Table 2. Figure 2a, illustrates the schematic 3D atomistic deposition of AlN on the Si substrate. The gray atomic slab represents the Si substrate, while the distribution of Al (depicted as cyan squares) and N (shown as blue squares) atoms indicates a layer-by-layer growth pattern. The position of each deposited atom was recorded, facilitating the analysis of the film morphology. Details regarding lattice parameters (a, b, and c), as well as vacancy and dislocation density, were derived from the positions of the deposited atoms within the lattice. The strain in each monolayer was calculated based on the extracted lattice parameters, layer by layer. Figure 2b illustrates the growth pattern of Al (cyan squares) and N (blue squares) atoms on the Si substrate pre-treated with NH_3_ for 30 s (represented by gray and blue squares). A notable difference was observed with the presence of an additional Si_3_N_4_ layer situated between the AlN and Si layers. The larger unit cell size of Si_3_N_4_ permitted the deposition of a greater number of Al and N atoms compared to the Si unit cell. This observation is corroborated by the total number of deposited atoms recorded for both samples, as detailed in Table 3.

**Fig. 3:**
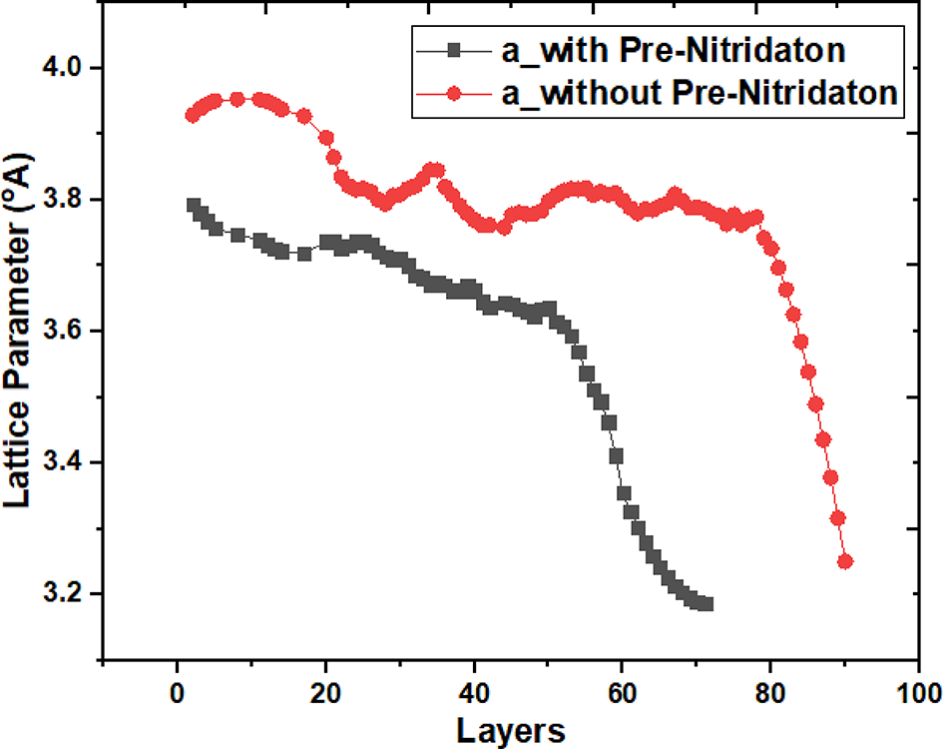
The change in the in-plane lattice parameter 'a' as a function of the total number of monolayers deposited on both the silicon and pre-nitridated silicon substrates.

A comparative analysis of the extracted lattice parameter ‘a’ for the AlN deposited layers was conducted on non-pre-nitridated (sample I) and pre-nitridated (sample II) substrates, as illustrated in Fig. 3. The lattice parameter ‘a’ for the AlN layers in both samples was determined to be approximately ~ 3.81 Å and ~ 3.84 Å, respectively. In contrast, the lattice parameters for Si and Si_3_N_4_ were measured at 3.84 Å and 7.603 Å, respectively. Notable variations in the lattice parameter ‘a’ for sample I were observed up to the 80 ML-AlN layers, which were attributed to strain resulting from lattice mismatch. After reaching 80 ML-AlN, a significant reduction in the lattice parameter was noted, as depicted in Fig. 3. This observation supports the findings in Fig. 1, indicating a transition in growth mode from 2D to layer by layer growth mode around the 80 ML-AlN layers in sample I. Conversely, sample II exhibited only minor variations in the lattice parameter up to the 50 ML-AlN layers, followed by a sudden decrease in the lattice parameter curve, indicating strain relaxation. It is a well-established phenomenon that strain tends to relax as the thickness increases. Therefore, the comparative study of the lattice parameters between samples I and II demonstrates that strain relief occurs within a lower critical thickness when utilizing an NH_3_ pre-flown substrate. While both curves exhibited a similar trend, the lower critical thickness identified in sample II provides valuable insights into the growth mechanism. The authors did not identify any precise experimental data regarding the lattice parameter as a function of layer-by-layer AlN deposition on a silicon substrate. Nevertheless, the results derived from the simulation study exhibit a consistent trend with the lattice parameters (a and c) of AlN epitaxy on SiC, as reported in the earlier experimental work by Nilsson et al.^29^. The in-plane lattice parameter of SiC is approximately 3.07 Å, while that of AlN is around 3.112 Å, according to the experimental findings in^29^. The experimental variation in the in-plane lattice parameter was noted to range from 3.10 Å to 3.12 Å. In the simulation study, the variation in lattice parameters was extracted as ranging from 3.8 Å to 3.2 Å and from 3.95 Å to 3.2 Å for AlN deposited on silicon (111) substrates, both without and with NH_3_ pre-flow, respectively. The significant variation in the lattice parameter of AlN, as illustrated in Fig. 3, can be attributed to the comparatively large lattice parameter of the Si substrate (3.84 Å), which exceeds that of SiC, as observed in the experiments. The significant variation in the extracted ‘a’ value for sample I can be attributed to the formation of SiN_x_ patches, as illustrated in Fig. 1a,b. Previous literature has elaborated on the complexities associated with the nucleation of AlN on the Si substrate^2,9,30,31^. The concurrent flow of TMAl and NH_3_ increases the likelihood of nitrogen presence across the entire Si (111) surface, in contrast to the aluminum from TMAl, due to the V/III ratio. Consequently, the likelihood of SiN_x_ formation, or nitrogen diffusion, is greater than that of aluminum diffusion into the Si (111) substrate. The probability of formation of SiN_x_ is dependent on the surface temperature. These SiN_x_ patches play a crucial role in enhancing the compressive strain within sample (I) Conversely, in the case of pre-flown NH_3_ substrate, the entire substrate surface is covered by Si_3_N_4_ layer with negligible probability of further nitrogen diffusion into Si substrate. The larger unit cell of Si_3_N_4_ in sample II appears to facilitate the deposition of aluminum and nitrogen atoms while minimizing the formation of SiN_x_ patches. Nearly two unit cells of AlN (a_ideal_ = 3.112 Å) can be accommodated within a single unit cell of Si_3_N_4_ (a_Si3N4_ = 7.603 Å). Additionally, the increased influx of aluminum atoms bonding with available sites on the pre-nitridated Si surface boosts the adsorption rate, as evidenced by the total number of AlN atoms deposited in each sample, with Total Deposited Atoms (AlN)_sample II_ exceeding Total Deposited Atoms (AlN)_sample I_, as shown in Table 3. The strain generation mechanism in each AlN layer was found to be less pronounced in sample (II) The generation of point defects (vacancies) was observed to depend on the strain on a layer-by-layer basis. Sample II exhibited a low vacancy density of approximately 0.2% relative to the total number of deposited atoms, while sample I showed a vacancy density of about 0.7%, as indicated in Table 3.

**Fig. 4:**
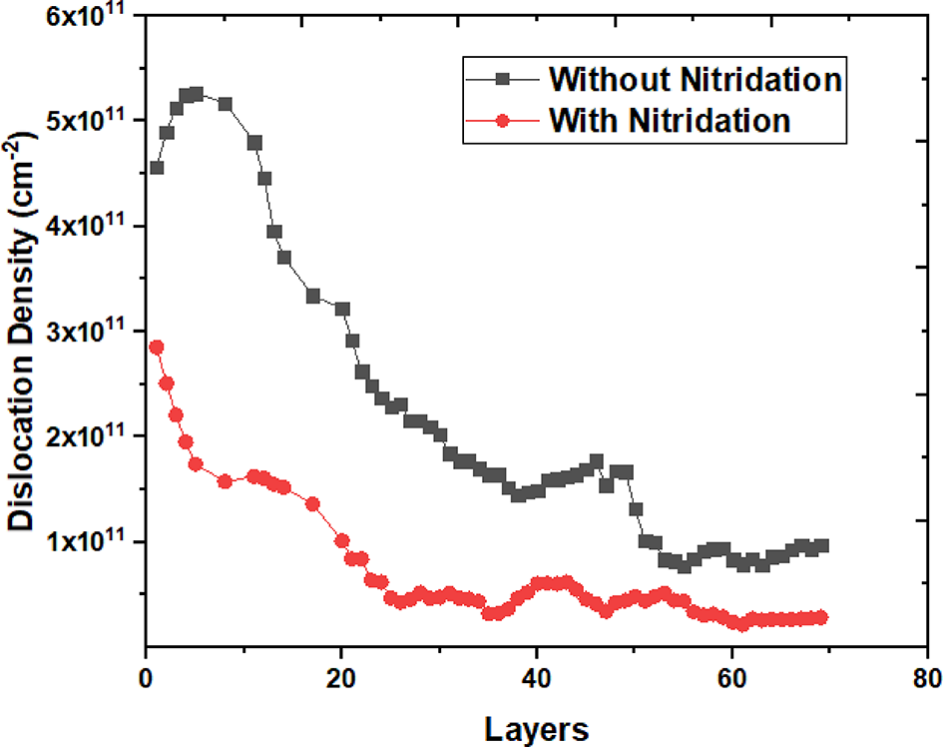
Dislocation density layer-by-layer AlN on Si(111) with NH_3_ pre-flow durations of 0s and 30s respectively.

The dislocation density data for samples I and II was analyzed and presented in Fig. 4. Sample I corresponds to the configuration without a pre-nitridated Si substrate, while sample II includes this pre-nitridation. In sample I, the dislocation density initially increased during the first 5 ML of AlN deposition, followed by a decrease until reaching a critical layer thickness of approximately 80 ML. The maximum dislocation density recorded for sample I was 5.3 × 10^11^ cm^− 2^, with a minimum of 9 × 10^10^ cm^− 2^. This elevated dislocation density can be attributed to discontinuities in the SiN_x_ interlayer, which generate strain, facilitating the nucleation of lower-quality AlN. Conversely, in sample II, the dislocation density peaked at the Si_3_N_4_/AlN interface, measuring around 3 × 10^11^ cm^− 2^, before declining as the critical thickness was approached. The minimum dislocation density observed in sample II was 2 × 10^10^ cm^− 2^. The reduced dislocation density in this sample is linked to the optimal NH_3_ pre-flow conditions. Adequate NH_3_ flow time ensures that nitrogen atoms are uniformly distributed across the Si (111) surface prior to the introduction of aluminum from TMAl, maximizing the likelihood of bond formation between aluminum and nitrogen atoms, while minimizing interactions with silicon atoms on the substrate. Consequently, the pre-nitridation process promotes the formation of a AlN single crystalline layer throughout the silicon wafer, resulting in a nitrogen layer adjacent to the silicon substrate and an aluminum layer above it, characterized by a lower dislocation density. The measured dislocation density values were consistent with those reported in previous experiments^19^.

**Fig. 5:**
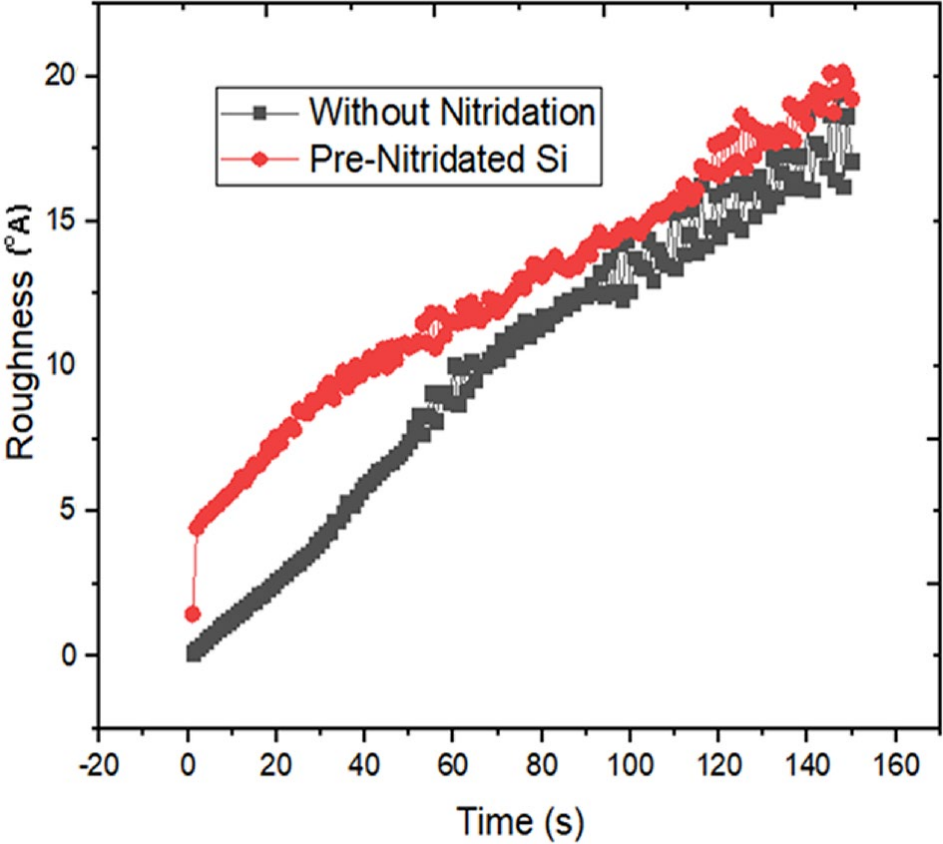
Analyzing the surface roughness in relation to the growth duration of AlN layers deposited on both silicon and pre-nitridated silicon substrates.

The roughness at each growth interval was determined by calculating the standard deviation of the height, where the height at each lattice point was assessed against the average height of all lattice points. The roughness values were significantly higher in sample II compared to sample I, as illustrated in Fig. 5. The substantial variation in roughness from the initial to mid-growth period is attributed to the favorable conditions that allowed a greater number of Al atoms to bond with N atoms across the entire Si (111) surface. The simulated roughness values align with the experimental findings, thereby confirming their validity^19^.

### Section II: multistep hetero-epitaxy

In the second case study, further simulations were performed to enhance the quality of GaN growth on Si (111) substrates by utilizing AlN and a three-step graded Al_x_Ga_1−x_N as buffer layers within a metalorganic chemical vapor deposition system^19^. The research meticulously analyzed the effects of varying NH_3_ pre-flow durations (0, 5, and 30 s) on the threading dislocation density (TDD) of the AlN, AlGaN strain relief layers, and GaN layers. Additionally, the investigation explored the development of compressive stress in the buffer layer and GaN films due to the differing NH_3_ pre-flow times.

Three samples were developed through atomistic simulation on Si (111) substrates using metalorganic chemical vapor deposition (MOCVD) within an AIXTRON CRIUS close-coupled-showerhead (CCS) reactor environment. The precursors employed were trimethylaluminium (TMAl), trimethylgallium (TMGa), and NH_3_, with hydrogen serving as the carrier gas. The pre-nitridation duration was adjusted for each of the three samples in increments of 0, 5, and 30 s at a temperature of 995 °C. The input parameters, as outlined in Table 4, were sourced from reference^19^. The growth process comprised six stages, starting with a low-temperature AlN layer of approximately 40 nm at 995 °C, followed by a high-temperature AlN layer of about 190 nm at 1095 °C, and then the Al_0.8_Ga_0.2_N (~ 235 nm), Al_0.5_Ga_0.5_N (~ 285 nm), and Al_0.2_Ga_0.8_N (~ 375 nm) layers at around 1070 °C, concluding with a 1 μm thick GaN layer at approximately 1050 °C. The mole fractions for all compositions were optimized prior to the simulation. The V/III ratios remained constant throughout each growth step for the respective layers, set at 280 for AlN, 1170 for Al_0.8_Ga_0.2_N, 760 for Al_0.5_Ga_0.5_N, 815 for Al_0.2_Ga_0.8_N, and 1550 for GaN. All layers in each sample were produced under a consistent reactor pressure of 1 × 10^4^ Pa, with growth rates aligned to those reported experimentally^19^.

**Fig. 6:**
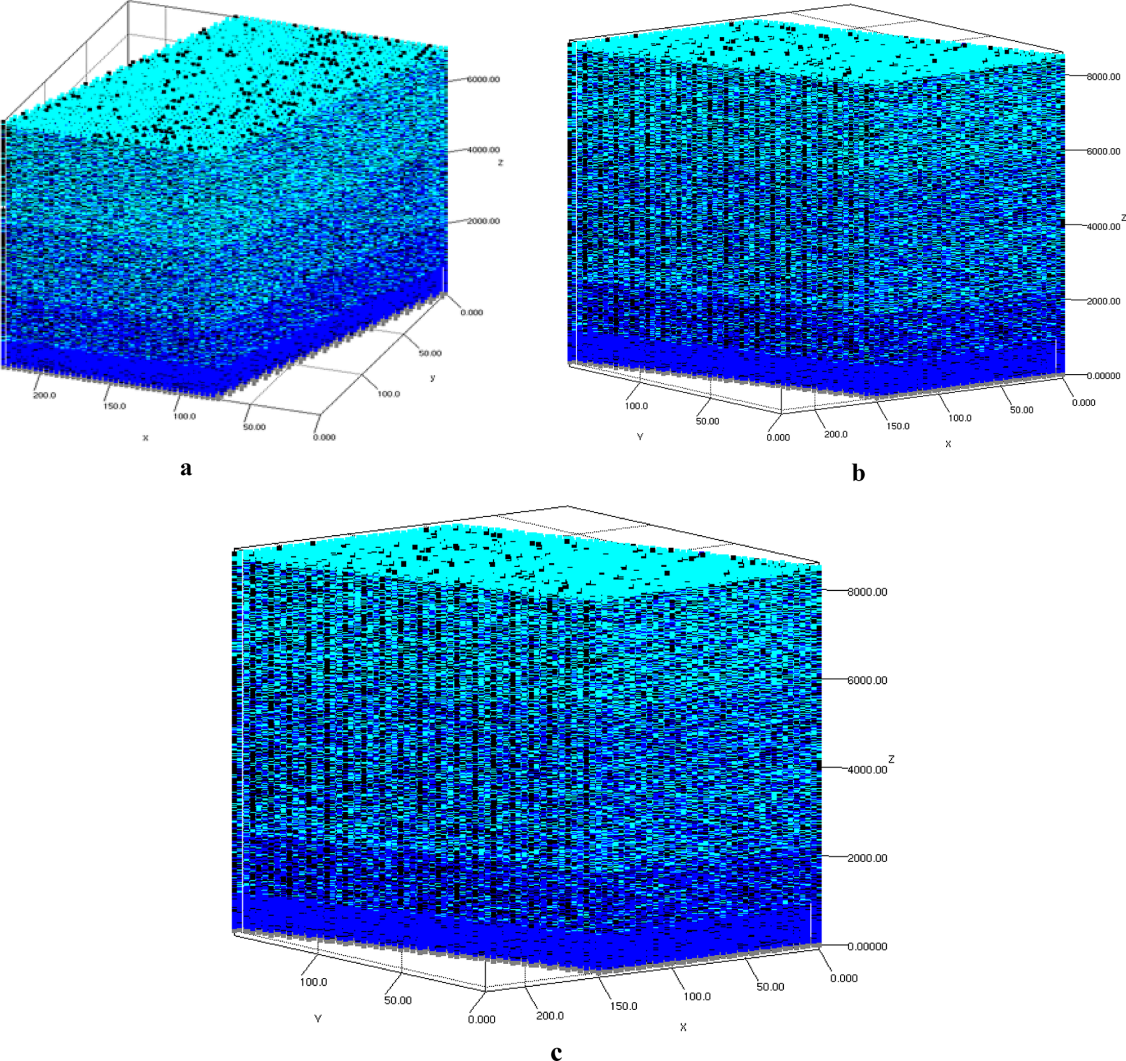
The three-dimensional atomistic distribution resulting from a seven-step epitaxy process encompasses low-temperature (LT) and high-temperature (HT) AlN, along with three graded Al_x_Ga_1-x_N strain relief buffer layers (where x equals 0.8, 0.5, and 0.2), followed by a GaN layer on a silicon substrate. This is illustrated for three different NH_3_ pre-flow durations: (**a**) 0 seconds, (**b**) 5 seconds, and (**c**) 30 seconds. In the representation, blue, cyan, and black squares denote Al, Ga, and N atoms, respectively, while Si is represented by gray squares.

**Fig. 7:**
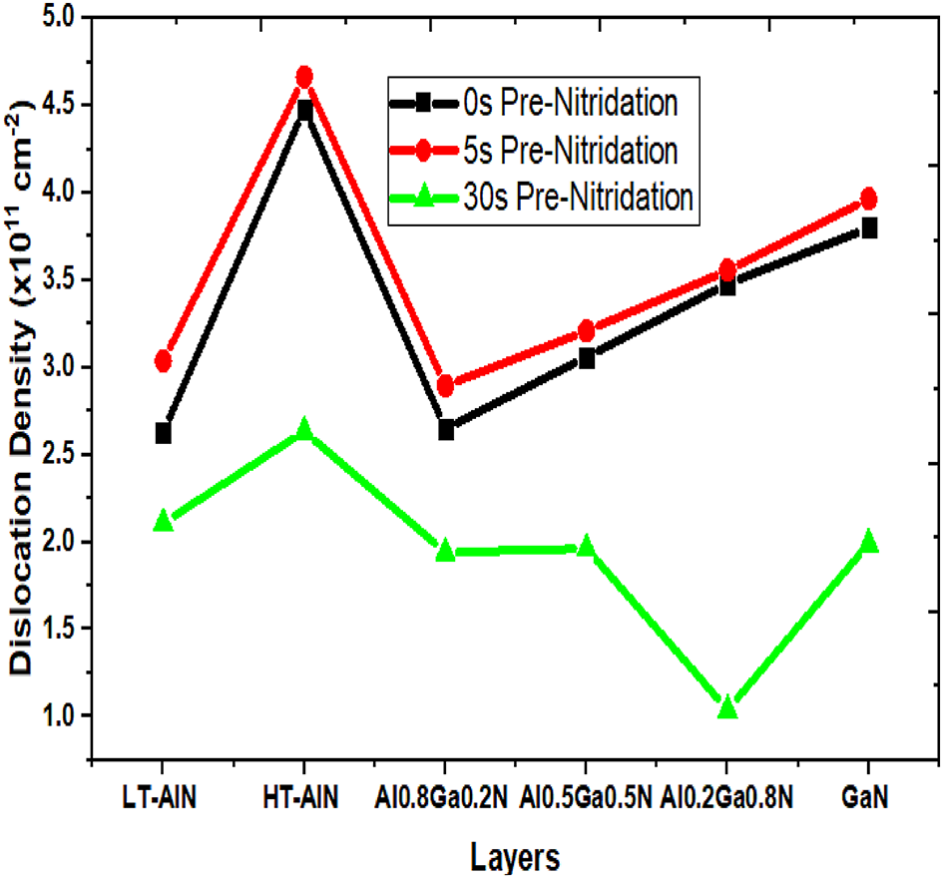
Analyzing the dislocation density profiles across each layer of deposited materials, specifically low-temperature (*LT*) and high-temperature (*HT*) AlN, along with three step-graded Al_x_Ga_1-x_N strain relief buffer layers (where x equals 0.8, 0.5, and 0.2), which are subsequently followed by GaN.

The 3D representations of three samples (A-pre-flow time 0s, B-pre-flow time 5s, C-pre-flow time 30s) are illustrated in Fig. 6a,b,c. The growth pattern, showcasing the atomistic arrangement of Al (blue squares), Ga (cyan squares), and N (black squares) atoms in relation to the Si atoms (gray squares), facilitated the extraction of information pertinent to each material layer and their interfaces. The positioning of each deposited atom within the lattice was analyzed to assess the film morphology. Detailed mappings of lattice parameters (a, b, and c), vacancy, and dislocation density were conducted to compare the effects of NH_3_ pre-flow time on growth morphology. It was noted that strain within each monolayer contributed to the formation of dislocation defects. The average dislocation density across the various material layers throughout the seven-step growth process for each sample (A, B, and C) is summarized in Table 5, while the comparative analysis of each sample across different material layers is presented in Fig. 7. The dislocation density data obtained in this study corroborates the results of the initial case study. Sample C, which underwent a 30-second pre-nitridation, demonstrated the lowest dislocation density when compared to samples A and B. In contrast, sample A exhibited a lower dislocation density than sample B. The introduction of NH_3_ for 5 s enhanced the likelihood of SiN_x_ patch formation, thereby increasing their density, which resulted in a slight rise in dislocation density throughout the LT AlN layer in sample B. The trends illustrated in Fig. 7 indicate a general increase in dislocation density across all three samples for the HT-AlN layer. The deposition of high-temperature AlN on the LT-AlN layer underscores the impact of elevated growth temperatures. The observed elongation of AlN atomic bond lengths due to thermal expansion during high-temperature deposition was identified as a factor contributing to increased strain, and consequently, a rise in dislocation density across all samples. In the subsequent growth phase, all three samples, now incorporating 20% Ga atoms, displayed a downward trend in dislocation formation. The addition of 20% Ga to AlN was found to reduce the TDD values, which can be attributed to the difference in atomic radii between Ga and Al atoms. With Al having a radius of 1.43 Å and Ga 1.35 Å, the 8% difference helps alleviate compressive strain to some extent. The TDD reduction for the Al_0.8_Ga_0.2_N layer was approximately 50% in samples A and B, while sample C showed a reduction of about 20% compared to the underlying HT-AlN layer. An increase in Ga content from 20 to 50% during the next strain relief phase resulted in a rise in TDD for samples A and B, while sample C remained unchanged. The increase in Ga atoms led to a higher TDD in samples A and B, whereas sample C maintained a constant TDD with 50% Ga content. The rise in the concentration of Ga atoms correlates with an increase in the threading dislocation density (TDD), as evidenced by the curves of samples A and B. However, in sample C, the TDD remained stable at a 50% Ga concentration. An increase in Ga content from 50 to 80% indicates a characteristic strain relief, resulting in a decrease in dislocation density for sample C. This suggests that a transition state was achieved, where the predominant compressive strain was transformed into tensile strain due to the elevated Ga content. The GaN deposition across all three samples (A, B, and C) exhibited a similar upward trend, supporting this conclusion. The TDD value extracted for sample C is approximately ~ 10^11^ cm^− 2^, which aligns with the experimental TDD value of 3 × 10^10^ cm^− 2^ obtained through high-resolution X-ray diffraction analysis, as referenced in^19^. The comparison between extracted average edge dislocation density values obtained via simulation against the experimentally HR-XRD values post annealing^19^ are depicted in Table 6. It is important to note that the TDD values were extracted immediately after the complete growth of the samples, while the experimental TDD measurement for the sample was conducted post-cooling and annealing process. Experimental samples undergo an additional annealing process at elevated temperatures before TDD measurement, during which recrystallization contributes to a reduction in dislocation density. The TNL-EpiGrow simulator currently does not offer the annealing or recrystallization feature. However, development of this functionality is in progress. Consequently, a slight difference of ~ 10x is observed between the simulated and experimental TDD values.

**Table 5:** Extracted dislocation density of IInd case study of part II.

Layers	A x 10^11^ cm^−2^	Bx 10^11^ cm^−2^	C x 10^11^ cm^−2^
LT-AlN	2.63	3.03	2.11
HT-AlN	4.48	4.66	2.63
Al_0.8_Ga_0.2_N	2.65	2.89	1.94
Al_0.5_Ga_0.5_N	3.06	3.20	1.96
Al_0.2_Ga_0.8_N	3.48	3.55	1.03
GaN	3.8	3.96	1.99

**Table 6:** Comparison between extracted average edge dislocation density against experimental edge dislocation density of IInd case study of part II.

Sample	Nitridation time (s)	TDD_Experiment_^19^	TDD_TNL-EpiGrow simulator_
I	0	1.2×10^10^	3.35×10^11^
II.	30	2.6×10^10^	1.9×10^11^

**Fig. 8:**
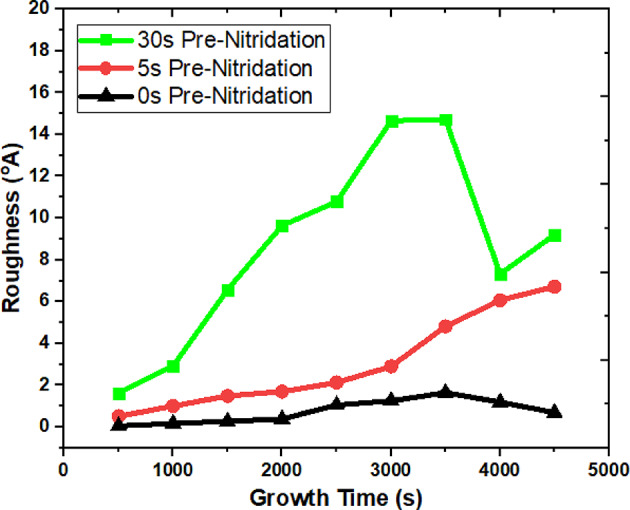
Variation in roughness over time of growth.

Figure 8 illustrates the surface morphology, specifically the changes in roughness over time. A comparison of the roughness curves for NH_3_ pre-flow times of 0s, 5s, and 30s indicates that all samples A, B, and C began with the same initial roughness value. It was noted that the root mean square (RMS) surface roughness for samples A and B, which underwent no nitridation or a nitridation time of 5s, remained low. In contrast, sample C exhibited an increase in roughness during the growth of AlN and AlGaN layers with Ga concentrations of 2% and 5%. The roughness curves derived from the growth simulation align with the experimental findings^19^. Additionally, it was found that the surface roughness of GaN with no nitridation (~ 0.07 nm) or with a shorter nitridation time (~ 0.6 nm) is lower than that observed with a nitridation time of 30s (~ 0.7 nm). The extracted RMS values are consistent with those obtained through experimental measurements^19^. Table 7 presents a comparison of the extracted RMS roughness values with those obtained through experimental methods^19^.

**Table 7:** Comparison between extracted RMS roughness with that of experimentally obtained of IInd case study of part II.

Nitridation time (s)	RMS roughness (experiment) (Å)	RMS roughness (TNL-EpiGrow simulator) (°A)
0	1.6	1.0
30	24.1	9.2

## Conclusion

A successful demonstration has been carried out to replicate the MOCVD epitaxy experiment of III-V nitrides on Si (111) substrates, employing atomistic simulation to assess the effects of NH_3_ pre-flow duration, or nitridation time. The findings revealed that the in-plane lattice parameter, strain, surface morphology (roughness), and threading dislocation density (TDD) were all affected by the length of the NH_3_ pre-flow time. The validity of the proposed approach was confirmed through the reproducibility of two separate case studies related to real-time experiments. An investigation into the formation mechanism of amorphous SiN_x_ patches indicated a strong dependence on nitrogen flux. It was found that various energy barriers—including nearest neighbor, surface, Schwoebel, incorporation, and desorption energy—were critical in influencing atom bonding and growth modes, which also impacted surface kinetics. The atomistic simulation method introduced is a cost-effective approach for epitaxy, providing opportunities to optimize different physical mechanisms with improved insights. Design engineers engaged in epitaxy processes can apply the guidelines offered, while the suggested simulation technique can be utilized to enhance new MOCVD reactor processes, ultimately resulting in decreased consumption of raw materials and carrier gases, thus reducing experimental costs.

## Data Availability

The data supporting the findings of this study are available from the corresponding author upon personal request.

## Code availability

All the results reported in current manuscript are obtained through TNL-EpiGrow simulator. Readers may reproduce the same output data using same input conditions. Download the TNL-EpiGrow simulator from www.technextlab.com for reproduction of results mentioned in the present manuscript.
